# Methods to Detect MHC-Specific IgE in Mice and Men

**DOI:** 10.3389/fimmu.2020.586856

**Published:** 2020-12-08

**Authors:** Anna Marianne Weijler, Jasmin Mucha, Andreas Michael Farkas, Ulrike Baranyi, Nina Pilat, Ara Cho, Moritz Muckenhuber, Stefan Hopf, Markus Wahrmann, Birgit Linhart, Rudolf Valenta, Thomas Wekerle

**Affiliations:** ^1^ Section of Transplantation Immunology, Department of Surgery, Medical University of Vienna, Vienna, Austria; ^2^ Cardiac Surgery Research Laboratory, Division of Cardiac Surgery, Department of Surgery, Medical University of Vienna, Vienna, Austria; ^3^ Department of Dermatology, Medical University of Vienna, Vienna, Austria; ^4^ Division of Nephrology and Dialysis, Department of Internal Medicine III, Medical University of Vienna, Vienna, Austria; ^5^ Division of Immunopathology, Department of Pathophysiology and Allergy Research, Center for Pathophysiology, Infectiology and Immunology, Medical University of Vienna, Vienna, Austria; ^6^ NRC Institute of Immunology FMBA of Russia, Moscow, Russia; ^7^ Laboratory for Immunopathology, Department of Clinical Immunology and Allergy, Sechenov First Moscow State Medical University, Moscow, Russia; ^8^ Karl Landsteiner University of Health Sciences, Krems, Austria

**Keywords:** transplantation, antibody-mediated rejection, donor specific antibodies, MHC, HLA, IgE

## Abstract

Humoral immunity is a major barrier limiting long-term outcome after organ transplantation. Especially, the production of antibodies directed against donor HLA/MHC antigens (i.e. donor-specific antibodies (DSA)) leading to antibody-mediated rejection (ABMR) is considered to be a major factor negatively affecting allograft survival. DSAs of the IgG isotype are routinely measured in transplant patients. However, not all patients diagnosed with IgG-DSA develop ABMR events. Therefore, research in better understanding the mechanisms of ABMR is of great importance. We recently demonstrated the production of MHC-specific IgE upon allograft rejection in mice and in transplant patients. IgE is classically connected with allergy and is known to be important for the humoral defense against helminths and worms. However, its role in autoimmune diseases and cancer has been reported recently as well. The concentration of IgE in blood is extremely low compared to other antibody isotypes. Therefore, detection of MHC-specific IgE from serum requires methods of high sensitivity. Since MHC-specific IgG—typically present at much higher serum levels—develops as well, high specificity is also required of IgE detection methods. In the murine model we developed an enzyme linked immunosorbent assay (ELISA) using MHC monomers for measurement of MHC-specific IgE, allowing us to distinguish between specificities of antibodies against different class I and class II antigens. For measurement of functional activity of MHC-specific IgE *in vitro*, a release assay using a rat basophil cell line (RBL-2H3) was established. For functional analysis of MHC-specific IgE *in vivo*, a cutaneous hypersensitivity reaction assay was adapted for this purpose using MHC monomers. Humanized RBL-2H3 cells transfected with cDNA coding for the human-high affinity IgE receptor were used for functionality measurement of donor-specific IgE in sensitized transplant patients. For detection of HLA-specific IgE, a bead assay was adapted, using beads expressing single HLA antigens. The aim of this publication is to demonstrate currently established methods for the detection and characterization of MHC-specific IgE in the murine and human setting.

## Introduction

For patients with end stage organ failure, transplantation is considered the treatment of choice. The 1 year survival rate after solid organ transplantation has increased significantly in the last decades, largely due to improvements in immunosuppression targeting T cell mediated rejection (TCMR). Research in improving long term outcomes, however, still faces huge challenges (1, 2). Amongst them the occurrence of antibody mediated rejection (ABMR), which is considered to be the leading cause for late graft loss, triggered by donor specific antibodies (DSA) against donor HLA or non-HLA targets (3–8). HLA-specific antibodies can either be pre-existing before transplant or develop *de novo* post-transplant. Donor specific antibodies of the IgG isotype are routinely measured in transplant patients (7). Although the occurrence of DSAs is considered to be a major risk factor for the development of ABMR, not all patients with confirmed levels of DSAs show histological signs of rejection (7, 9). Diagnosis and treatment of ABMR are challenging, as the underlying pathological mechanisms are hitherto incompletely understood (10, 11). Therefore, more insight about the key mechanisms of humoral rejection is urgently needed. Studies in the field of humoral rejection focus mainly on DSA of the IgG isotype, as it is considered to be the most important one, with few publications about HLA-specific IgA and IgM (3, 10, 12).

We recently demonstrated the development of donor specific antibodies of the IgE isotype in mice and highly sensitized kidney transplant patients (13). Antibodies of the IgE isotype are mainly connected with inflammatory immune responses of the Th2 type, as in allergy, and infections with parasites, such as worms or helminths (14, 15). Furthermore, a potential role of IgE auto-antibodies in diseases like atherosclerosis, lupus and atopic dermatitis has been shown (16–19). More importantly, recent insights in the role of IgE not only in Th2 driven immune responses, but also as protective factor in skin cancer leads to the assumption that the influence or role of IgE in the immune system might be much wider than previously thought, including the field of transplantation immunology (20). Several studies indicate that the Th2 response is important for the development of graft injury. It was shown that within the graft the appearance and degranulation of IgE effector cells, i.e. basophils, mast cells and eosinophils, play a significant role in transplant rejection (21–26). Therefore, defining the role of MHC-specific IgE in transplant rejection might be a further step in a better understanding of the pathological mechanisms of ABMR.

Levels of IgE in serum are extremely low compared to antibodies of the IgG isotype (1:10,000). In fact, with plasma concentrations levels of less than 1 µg/ml in healthy humans, a new standard unit (kU/L or IU/ml) was presented in 1981 by ‘The National Institute for Biological Standard and control’ in order to properly express the concentration of serum IgE (27). Additionally, free circulating IgE in blood has a very short half-life, compared to antibodies of other isotypes (2 days vs. 21 days for IgG) (28). Due to the before mentioned very low plasma concentration levels in combination with the short half-life of IgE very sensitive and highly specific methods to detect and quantify antigen-specific IgE are required (14). For our research on the role of MHC-specific IgE in humoral rejection, we adapted a method used in allergy research, the allergen-specific ELISA (29). By using recombinant murine MHC-monomers it is possible to determine MHC-specificity of donor reactive IgE *via* a MHC-specific ELISA. It is known, that DSA directed against MHC class II are clinically relevant predictors of ABMR (30). As by now, standard procedure of DSA detection in the murine model is limited to measure either DSAs against MHC class I using donor thymocytes as targets or DSAs against MHC class I and class II using donor B cells *via* flow crossmatch. This method, however, does not allow to determine the antigen-specificity of the detected DSAs. With the use of the newly established ELISA it is possible to determine antigen specificity. To our knowledge, this assay represents a unique way of DSA detection in the murine setting through the use of MHC monomers and additionally offers the possibility to detect DSA of other isotypes as well.

Free IgE has a shorter half-life compared to the IgG antibody isotype and is stabilized *via* binding to its effector cells like basophils or mast cells. Once IgE is bound to its effector cells it can lead to inflammation and tissue destruction. By binding to its high affinity receptor FcϵRI on basophils or mast cells and subsequent crosslinking through the specific antigen, cell degranulation is induced releasing inflammatory mediators such as histamine, proteases, heparin and various cytokines (31). In extreme cases of allergy, this can lead to anaphylactic shock (32, 33). In the case of transplantation immunology, IgE might play a role in the development of ABMR. To prove the functional activity of allergen-specific IgE in allergy research a basophil release assay is typically performed using a basophilic leukemia cell line (RBL-2H3) (34). RBL-2H3 cells are incubated with the serum followed by incubation with the respective allergen. Cell degranulation is induced, if allergen-specific IgE is present. Percentage of degranulation is calculated according to the release of an enzyme called β-hexasominidase. Through the adaption of this RBL-2H3 assay, it is possible to test if MHC-specific IgE is functional by showing its potential to initiate degranulation *in vitro* using MHC monomers as specific antigen *(* 34 *)*. For functional analysis of MHC-specific IgE *in vivo*, we adapted the cutaneous type I hypersensitivity reaction assay for our purposes (35). Evans Blue is injected intravenously into a sensitized mouse followed by an intradermal injection of MHC monomers. Vasoactive mediators are released due to mast cell degranulation, leading to an increased vascular permeability. A blue discoloration of the skin can be seen, provided that functional mast cell-bound MHC-specific IgE is present.

Elevated numbers of IgE antibodies in transplant patients were already suggested in 1980 by a Spanish research group (36). With the use of a radioimmunoassay they showed elevated numbers of IgE molecules on the surface of basophils isolated from kidney transplant patients. We adapted a methodology for the detection of HLA-specific antibodies routinely used in clinics for DSA screening. By using single antigen beads in a bead-based multiplexed immunoassay system (e.g. Luminex^®^), it is possible to identify antibodies binding to an array of defined HLA- antigens in a human serum sample by a single multiplex test (37, 38). Such assays are routinely used in the clinical transplant setting to screen for HLA-specific IgG antibodies already before and after transplantation. We established a SAB Luminex assay for detection of HLA-specific IgE for research purposes. Moreover, we successfully downscaled the bead volume as an optimization for research use with respect to the limitations of the assay. To demonstrate functional activity of HLA- specific IgE antibodies, we established a humanized RBL-2H3 assay analogous to the assay for the murine setting. Using RBL cells transfected with cDNA coding for the human high affinity IgE receptor chains α, β we could show HLA- specific IgE functionality by degranulation and mediator release *in vitro* (39).

This publication describes recently established methods for the detection of MHC-specific IgE in mice and humans and the demonstration of its functional activity. Screening for IgG-DSAs is standard in clinical routine diagnostics. Detection of other DSA isotypes, including now IgE, is a tool to get more insight into the humoral allograft response. These methods are particularly valuable for research groups in the field of transplantation immunology.

## Materials and Methods

### Mice

Female C57BL/6 (haplotype H-2b) and BALB/c (haplotype H-2d) mice were purchased from Charles River Laboratories, Sulzfeld, Germany. Female C3H/J (haplotype H-2k) mice were purchased from Jackson Laboratory (Bar Harbor, ME) and bred at the Department of Biomedical Research, Medical University of Vienna (Austria). Mice were kept in a protected environment and a 12-h light-cycle in individually ventilated filter cages on sterile standard beddings. Sterile water and standard pellet diet were given ad libitum. Mice between the ages of 6 to 8 weeks with an average weight of 18 to 22 g were used for the experiments.

### Skin Grafting

Mice were anesthetized with ketamine (Ketalar, 100 mg/kg) and xylazin (Rompun, 5 mg/kg) in 0.9% NaCl. Full thickness tail skin from BALB/c (donor) or C3H (donor) mice was grafted on the left side of the chest and secured with stitches and with band aids for 7 days. Postoperative analgesia consisted of piritramid (Dipidolor, 15 mg) and 0.4% glucose in 250 ml drinking water ad libitum for one week. Grafts were visually inspected every day. They were considered rejected if less than 10% of the graft remained viable. Skin grafts from fully mismatched donors without special immunosuppressive treatment of the recipients were rejected about 10 days after transplantation.

### ELISA for MHC-Specific IgE Detection in Mice

**Figure 1:**
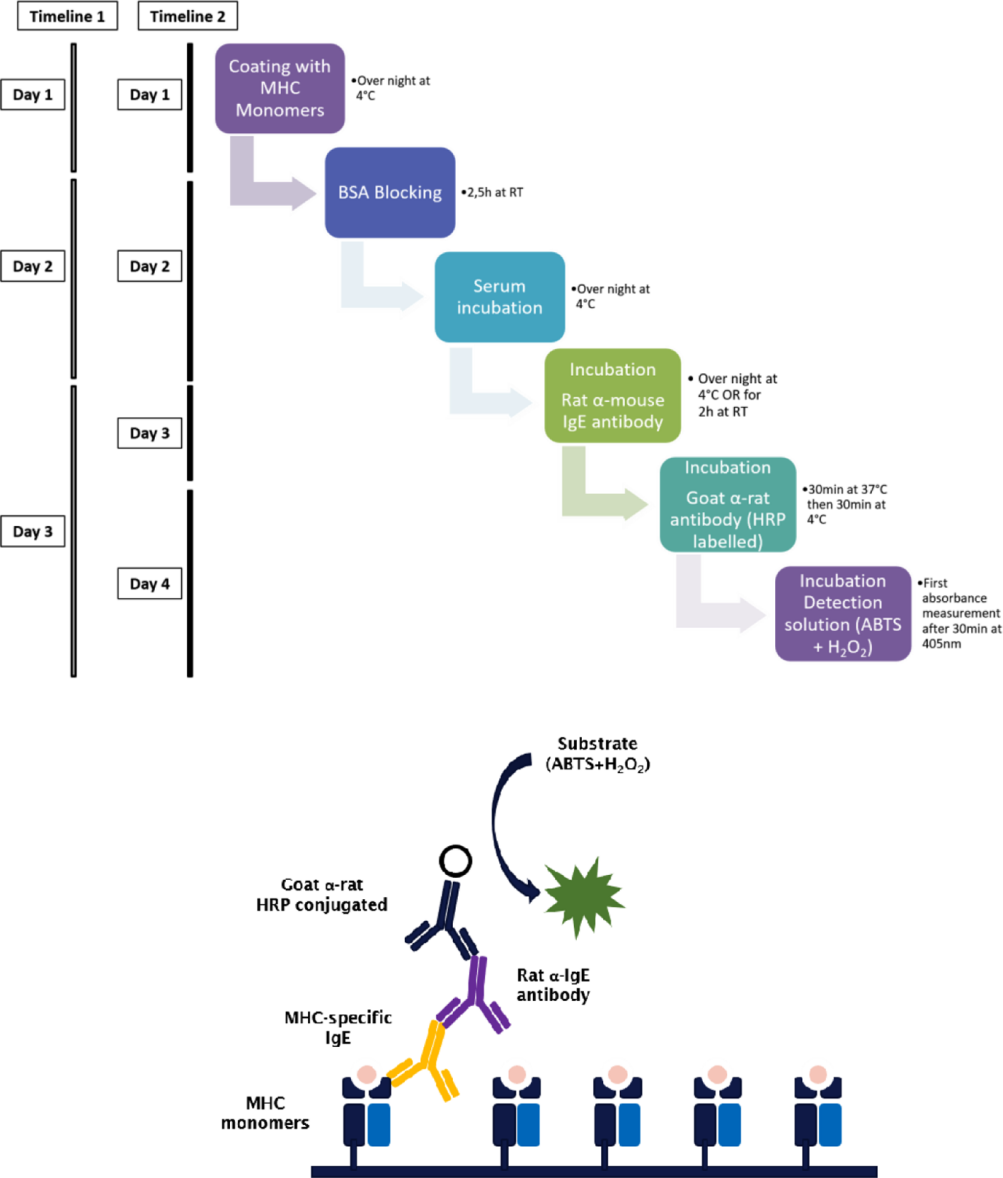
Step by step procedure and schematic representation of the MHC-specific ELISA. Murine MHC monomers are incubated with murine serum to be analyzed for the presence of MHC-specific IgE. MHC-specific antibodies bind to their respective epitope on the monomers and are then detected using a primary antibody. The timeline of the ELISA procedure varies with the incubation time and temperature of the primary antibody. When incubated for 2 h at RT the ELISA takes three days (timeline 1), when incubated at 4°C overnight, the ELISA takes four days (timeline 2). The secondary or detection antibody is conjugated with HRP. Due to the presence of hydrogen peroxide and HRP, ABTS is oxidized to a radical cation. The resulting blue-green reaction product of ABTS can be measured at 405 nm.

A maximum absorbance 96-well plate was incubated with 5 µg/ml (100 µl of per well) of the respective monomeric MHC antigen diluted in coating buffer (3.9 g Na_2_CO_3_, 5.3 g NaHCO_3_ in 1000 ml ddH_2_O, pH 9.5) over night at 4°C. Plates were washed twice with washing buffer (PBS/0.05% Tween-20). For saturation of unspecific binding sites, the plate was incubated with PBS/1%BSA/0.05% Tween-20 at room temperature (RT) for 2.5 h, followed by five washing steps with washing buffer. Mouse serum was diluted with dilution buffer (PBS/0.5% BSA/0.05% Tween-20) 1:2.5 for IgE or 1:125 for detection of IgG1. The plate was incubated with 100 µl/well of the respective serum dilution over night at 4°C. After washing of the plate five times using the washing buffer, bound antibodies were detected using monoclonal rat α-mouse IgE (clone R35–72) or α-mouse IgG1 (clone A85-1) (BD, San Diego, CA, USA) diluted to 0.5 µg/ml in dilution buffer (100 µl/well). After incubation for 2 h at RT or at 4°C overnight, and a further washing step with washing buffer, the plate was incubated for 30 min at 37°C and 30 min at 4°C with HRP-coupled goat anti-rat serum (Amersham, Biosciences, UK) diluted 1:2000 with dilution buffer (100 µl/well). ABTS (60 mM/L citric acid, 77 mM/L Na_2_HPO x2H_2_O, 1.7 mM/L ABTS [Stigma, St. Louis, MO, USA], 3 mM/L H_2_O_2_) is used as the detection substrate for HRP. This solution has to be prepared freshly and H_2_O_2_ is added just before detection. An Infinite microplate reader (Infinite F50, TECAN) was used for absorbance measurements at 405 nm. Background measurement was recorded at 490 nm (**Figure 1**).

MHC I H-2K^k^, MHC I H-2K^d^, MHC I H-2D^k^, MHC I H-2D^d^, MHC II I-E^k^, MHC II I-E^d^ MHC II I-A^k^, MHC II I-A^d^ monomers were kindly provided by the NIH Tetramer Core Facility (https://www.niaid.nih.gov/research/nih-tetramer-core-facility).

### Rat Basophil Leukemia Cell Degranulation Assay for *In Vitro* Functional Assessment of MHC-Specific IgE

**Figure 2:**
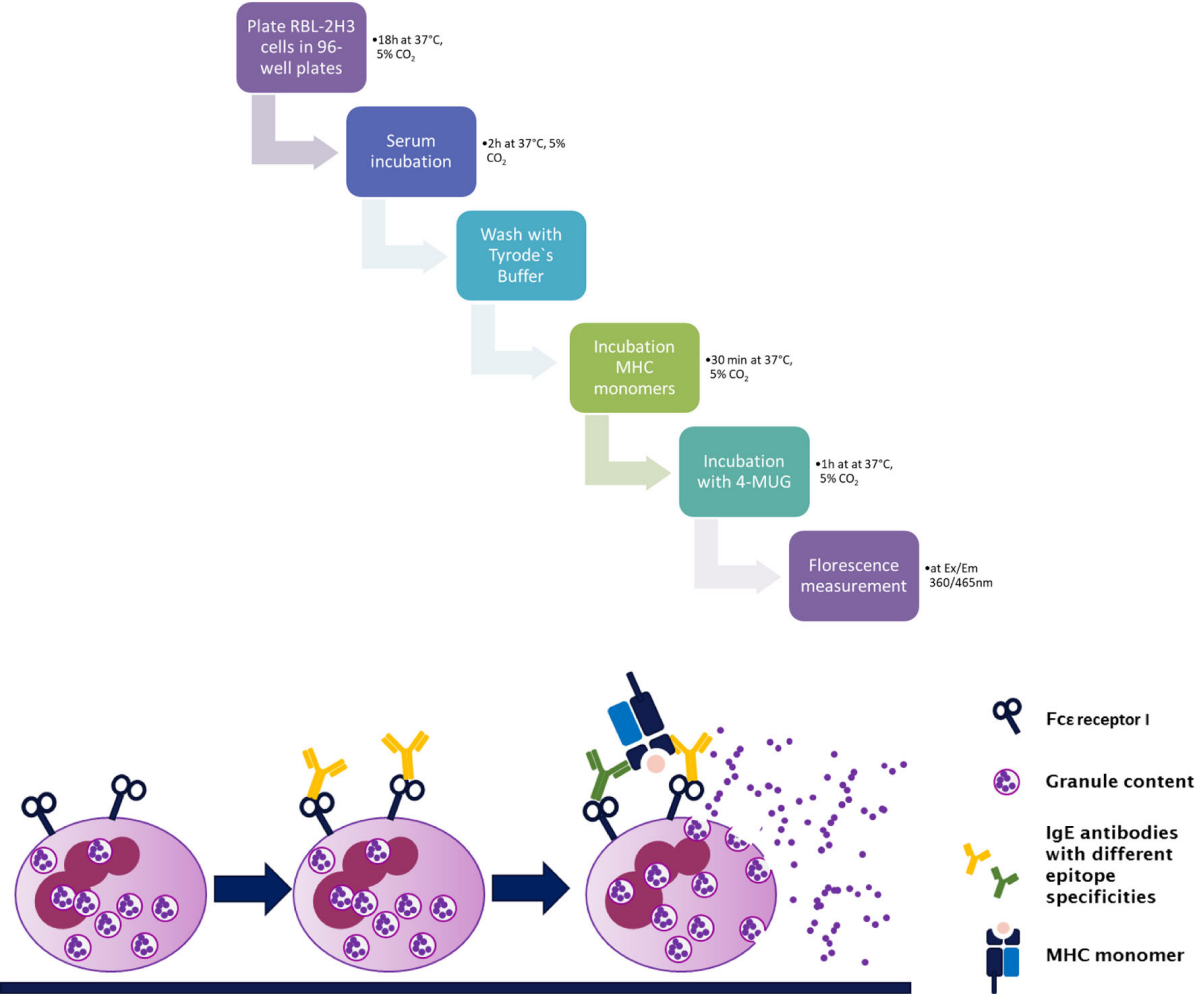
Step by step procedure and schematic representation of the rat basophil leukemia cell degranulation assay. Cells from the rat basophilic leukemia strain (RBL-2H3) are incubated with murine serum that shall be tested for the presence of functional MHC-specific IgE. MHC-specific IgE binds to its high affinity receptor FcϵRI. This is followed by incubation with a murine MHC monomer (class I or class II). Crosslinking of bound IgE antibodies *via* specific antigen, in this case MHC, leads to degranulation of the RBL-2H3 cells, releasing β-hexasominidase, amongst other granule content. The detection substrate 4-MUG is cleaved to 4-MU *via* this enzyme leading to a fluorescence signal at Ex/Em 360/465 nm.

Cells from the RBL-2H3 subline were cultured in RPMI 1640 medium (Biochrom, Berlin, Germany) with 10% FCS. Upon start of the assay, 4 × 10^4^ cells were plated on a 96-well tissue culture plate (Greiner Bio-One, Kremsmünster, Austria) and incubated for about 18 h at 37°C, 5% CO_2_. Cells were incubated with mouse serum diluted 1:2.5 with medium (100 µl/well) for 2 h at 37°C, 5% CO_2_ followed by a washing step with 2× Tyrode’s buffer (9.5 g Tyrode’s Salts [T2145, Sigma-Aldrich, St. Louis, MO], 2.38 g 10 mM HEPES, 1 g NaHCO_3_, 0.1% BSA in 1,000 ml ddH_2_O). Next, the cell layer is incubated with 3 ng/well of a monomeric MHC antigen (H-2K^k^, H-2K^d^, H-2D^k^, H-2D^d^, I-E^k^, I-E^d^, I-A^k^, or I-A^d^, respectively; NIH tetramer Core Facility), for 30 min at 37°C. At least three wells of cultured cells were spared of serum and antigen treatment and were used as positive control for calculation of the percentage of antigen-specific degranulation of the RBL-2H3 cell line. They were incubated with 1% Triton X-100, which leads to cell death and therefore to a maximum (100%) release of β-hexasominidase (**Equation 1**). The analytic compound 4-methylumbelliferyl-*N*-acetyl-β-d-glucosamide (4-MUG; Sigma-Aldrich, St. Louis, MO) is aliquoted beforehand to 10 mM stock solutions in DMSO for storage at −80°C. For measurements of the β-hexasominidase release of degranulated basophils, 80 µl of 4-MUG stock solution is transferred to 5 ml of citrate buffer (19.2 g 0.1 M citric acid, or sodium citrate, in 1,000 ml ddH_2_O; pH 4.5) and the plate was incubated with this assay solution for 1 h at 37°C. Glycine buffer (15 g Glycine**;** 11.7g NaCl in 1,000 ml ddH_2_O; pH 10.7) was used to stop this reaction and the fluorescence was measured using an Infinite plate reader (Infinite F50, TECAN) at *λ*Ex: 360/*λ*Em: 465 nm (**Figure 2**).

**Equation 1** Calculation of the percentage of antigen-specific degranulation of the RBL-23H cells. RFU, relative fluorescence units

$$\frac{{\left[RFU\right]}_{MHC\;monomer}}{{\left[RFU\right]}_{TritonX100}}*100=\%\;RBL-2H3\;degranulation$$

### Cutaneous Type I Hypersensitivity Reaction for *In Vivo* Functional Assessment of MHC-Specific IgE

BALB/c mice were injected intravenously (i.v.) with 100 μl 0.5% Evans Blue (Sigma-Aldrich, St. Louis, MO). 30 μl of MHC monomer (rH-2K^k^ or rH-2D^k^, 2 μg/ml diluted with PBS) were injected into the shaved abdominal skin. 30 µl of the mast cell-degranulating compound 48/80 solution (20 µg/ml, Sigma-Aldrich, St. Louis, MO) was injected intra-dermally as positive control. As negative control, PBS was used. After 15 min, mice were sacrificed and their inverted skin was mounted to a foamed polystyrene plate and documented by photography. The degranulation of mast cells, due to crosslinking of bound IgE with the specific antigen, leads to a release of vascular mediators. These mediators increase the permeability of ambient vascular structures. Previously injected Evans Blue is then released into the surrounding tissue leading to a blue discoloration of the skin. Blue discoloration around the respective injection site indicates the presence of MHC-specific IgE against the respective injected MHC monomer.

### Human Serum Isolation

Whole blood was collected in a serum tube containing clot activator and stored by RT for 30 min waiting for blood coagulation. Afterwards the tube was centrifuged for 10 min at 1500 x g at 10°C. After centrifugation serum was aliquoted and stored at −80°C.

### Bead-Based Assay for HLA-Specific IgE Detection in Humans

**Figure 3:**
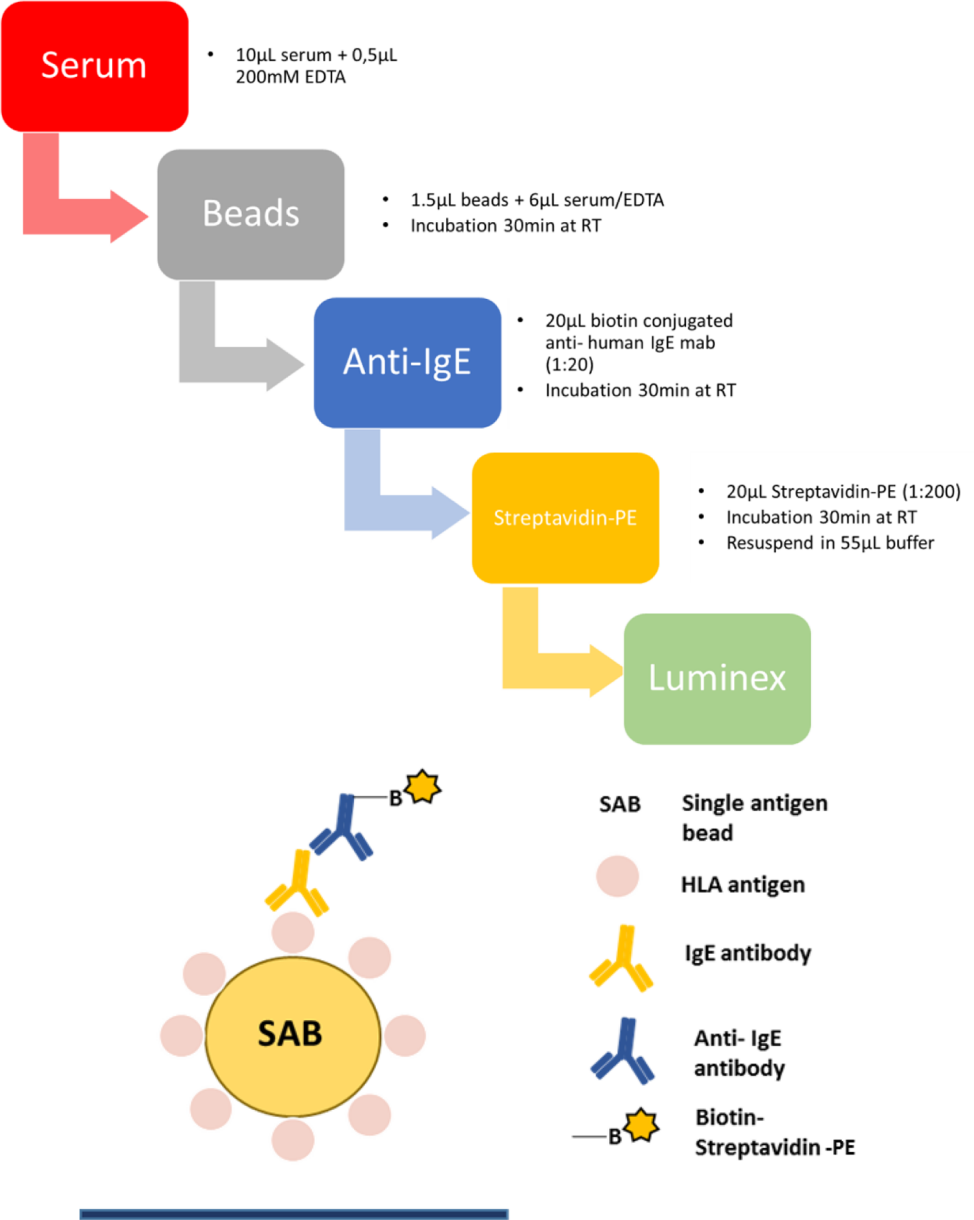
Step by step protocol of detection HLA-specific IgE in human serum with a luminex flow analyzer. Human serum sample was mixed with 10 mM EDTA and incubated with beads coated with single antigens at RT. After incubation, beads were washed twice with washing buffer and incubated with a monoclonal biotin conjugated anti-human IgE antibody. Beads are washed again twice, phycoerythrin (PE)-coupled streptavidin was added and after incubation and repeated washing steps beads were resuspended in 55 µl washing buffer and measured in a Luminex instrument.

*One Lambda SAB* kits (LABScreen Single Antigen HLA class I, #LS1A04 and HLA class II, #LS2A01 antibody detection tests) were used to investigate HLA- specific IgE in patient serum and to perform downscaling experiments of the beads. Patient serum was adjusted to 10 mM EDTA (Invitrogen) to prevent complement interference phenomenon (40, 41). Next, 6 µl serum and 1.5 µl beads were incubated in a 96 well V-bottom plate (Merck, Microplate Devices Uniplate) for 30 min at RT in the dark and on a shaker at 550 RPM. The plate should be vortexed every 10 min. In the following step, beads were washed twice with washing buffer (OneLambda, 10× diluted 1:10 with ddH_2_O) by centrifuging at 1800 x g for 7 min at 4°C and incubated with biotin-conjugated anti- human IgE mab (Biolegend, clone MHE-18) at a dilution of 1:20 (0.5 µg/well) for 30 min in the dark on a shaker. In the next step, beads were washed twice and PE-conjugated streptavidin (eBioscience) at a dilution of 1:200 (0.02 µg/well) was added and the incubation step was repeated. After incubation, samples were washed twice again, beads were resuspended in 55 µl buffer and then measured on the *Luminex flow analyzer 200*. The protocol is schematically illustrated in **Figure 3**. The cut-off for a positive anti- HLA IgE signal was defined as the mean of the median fluorescence intensity (MFI) of three healthy donors plus 2× standard deviation (SD) (Equation 2) (13, 15). Additionally, only a minimum MFI of >25 was counted as positive. Several considerations informed the definition of this preliminary threshold. In the basophil release assay, described below, degranulation was only observed if the MFI in the Luminex assay was at least ≈25 for a given antigen specificity. Results from healthy male volunteers without a history of transplantation or blood transfusion, provided negative control values, whose mean + 2*SD was set as boundary to positive values.

**Equation 2**: Threshold definition for positive HLA-specific IgE signals. For calculation of MFI threshold measurements of healthy male donors were used. SD, standard deviation; MFI, median fluorescent intensity.

Threshold=Mean MFI healthy donors + 2∗SD

Threshold positive signal > MFI 25

### Humanized Rat Basophil Leukemia Cell Degranulation Assay for *In Vitro* Functional Assessment of HLA-Specific IgE

To test functionality of HLA-specific IgE antibodies *in vitro*, a mediator release assay with humanized rat basophil leukemia (RBL-2H3) cells (clone RBL-703/21) was performed (13). In brief, RBL-2H3 cells transfected with human cDNA coding for the high affinity IgE receptor chains α, β and γ were cultured in RPMI 1640 medium (Biochrom, Berlin, Germany) with 5% FBS, 4 mmol/L l-glutamine, 1 mg/ml gentamicin sulfate at 37°C and 5% CO_2_ and plated in 96 well plates (1 × 10^5^ cells/well) in technical triplicates. The cells were incubated overnight together with sera from highly sensitized kidney transplant patients and as a control with sera from healthy subjects. Beads coated with a single HLA- antigen were used (LS Single, One Lambda, Canoga Park, CA, USA). An HLA antigen was selected to which the patient showed a positive IgE signal in the Luminex assay and one to which he/she was negative on Luminex as a specificity control. Beads were incubated with RBL-2H3 cells and human serum for 1 h at 37°C. The reaction was stopped with 100 µl glycin buffer and basophil degranulation was measured by β-hexosaminidase release by an Infinite plate reader (infinite F50, TECAN) at *λ*Ex: 360/*λ*Em: 465 nm.

### Statistics

Murine data were statistically analyzed using GraphPad Prism 8.0 (Graph Pad Inc., La Jolla, CA). P values were calculated using the 2-sided Mann-Whitney U test. A p value lower than 0.05 was considered statistically significant (* p < 0.05, ** p < 0.01)

## Results

### Detection of MHC-Specific IgE in Mice

The detection of allergen-specific IgE using an ELISA is common in allergy research (29). The established assay for the measurement of MHC-specific IgE was based on this method. Antibodies of the IgE isotype are present at very low concentrations in serum compared to antibodies of the IgG isotype (1:10,000) (14). Important steps of this method had to be adapted to increase the sensitivity without losing the specificity for detection of donor-specific IgE.

Allergen-specific IgE in isolated murine and human serum has shown to be stable for a period of time also on RT (42). However, murine serum samples used in the establishment of these methods were kept on ice at all times during preparation and were thawed as close to the start of the experiment as possible. For long-term storage, serum was kept at −20°C and was not thawed more than two times.

**Figure 4:**
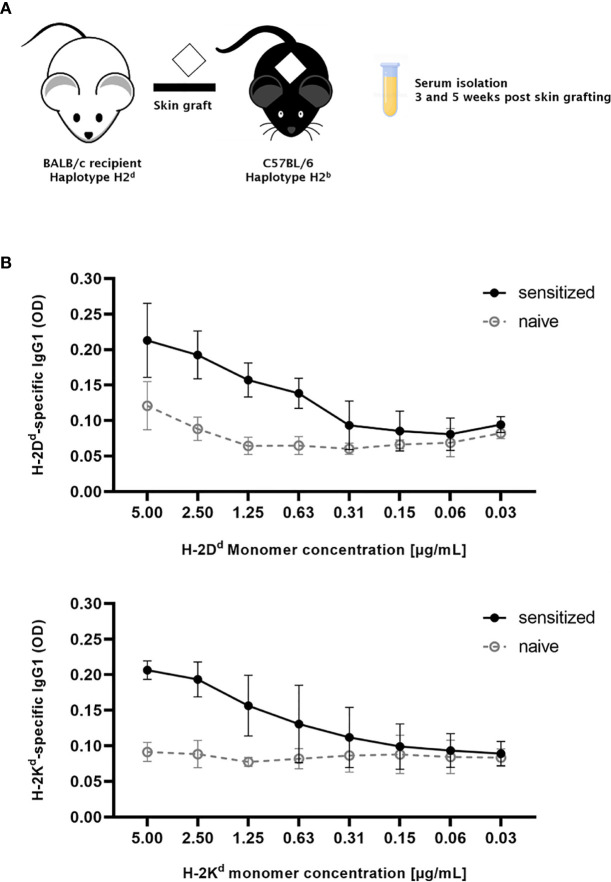
Titration of MHC monomers. **(A)** Schematic illustration of the experimental set up. C57BL/6 mice were grafted with fully mismatched BALB/c skin grafts. Serum was isolated 3 and 5 weeks post skin grafting. **(B)** For detection of the appropriate monomer concentration, plates were coated with MHC class I monomers (H-2D^d^, H-2K^d^) diluted from 5 to 0.03 µg/ml, followed by incubation with either sensitized serum from C57BL/6 mice after rejection of a BALB/c skin allograft, or with naive serum as negative control. Results from two independent experiments are shown. Serum in each experiment was pooled and individual values were measured in triplicates.

As a first step, MHC-monomers were titrated using an antibody against IgG_1_. 96-well plates were coated with MHC class I (H-2K^d^, H-2D^d^) monomer concentrations ranging from 0.03 to 5 µg/ml (**Figure 4**). The clearest signals of MHC-specific IgG_1_ in sensitized serum compared to naïve serum were achieved at the highest monomer concentration (5 µg/ml), without a significant increase in background signal. To ensure binding and therefore detection of IgE despite high levels of donor-specific IgG_1_ potentially binding to the same MHC epitopes, the highest concentration of MHC monomers was thus selected for use in all further experiments.

**Figure 5:**
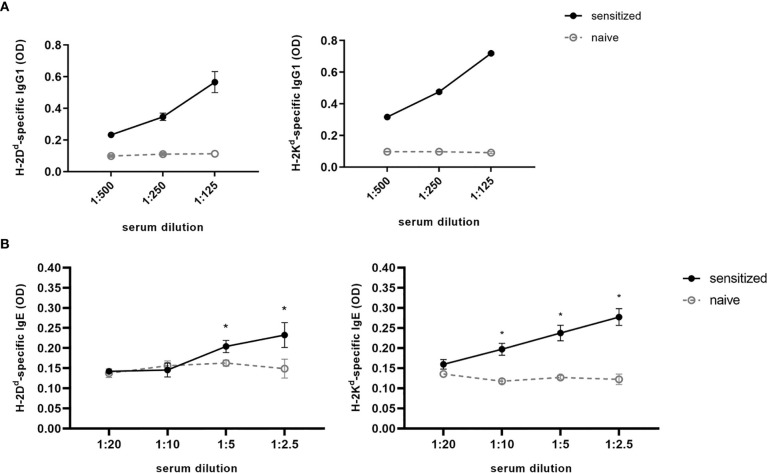
Serum titration for detection of MHC-specific IgG and IgE. For H-2D^d^- or H-2K^d^-specific IgG_1_ **(A)** or IgE **(B)**, a serial dilution of serum isolated from C57BL/6 mice sensitized with a BALB/c skin graft was performed. Serum dilution is ranging from 1:500 to 1:125 or from 1:20 to 1:2.5 for IgG_1_ and IgE, respectively. As negative control naive C57BL/6 serum was used. Results from two independent experiments are shown for MHC-specific IgE (n=4 for sensitized and naïve). P values were calculated using the Mann-Whitney U test *P < 0.05. Different dilutions of MHC-specific IgG1 were shown in one experiment (n=2 for sensitized and naïve).

Although levels of MHC-specific IgE were very low, serum had to be diluted for detection, as using undiluted serum leads to high unspecific background signals. Different serum dilutions for detection of MHC-specific antibodies are shown in **Figure 5**. The most distinct difference between naïve and sensitized serum for the detection of MHC-specific IgE was achieved by using a dilution of 1:2.5 with serum dilution buffer, which was also used for our further experiments. For detection of MHC-specific antibodies of the IgG isotypes, a serum dilution of 1:125 was used.

The specificity of this method of detection was verified by incubating MHC monomers (H-2K^d^, H-2D^d^) with serum of different recipient and donor strain combinations. A positive signal was only expected for C57BL/6 mice after rejection of a BALB/c skin allograft (haplotype d). Serum was isolated three and five weeks after rejection and diluted, as mentioned before, 1:2.5 using PBS/Tween/BSA. Positive signals were only detected against monomers of the haplotype d. No positive signals were seen after incubation with serum of C57BL/6 mice or BALB/c mice sensitized with C3H skin allografts (haplotype k), demonstrating the specificity of this method to detect MHC-specific IgE [for these specificity data see Figure E2 in (13)].

**Figure 6:**
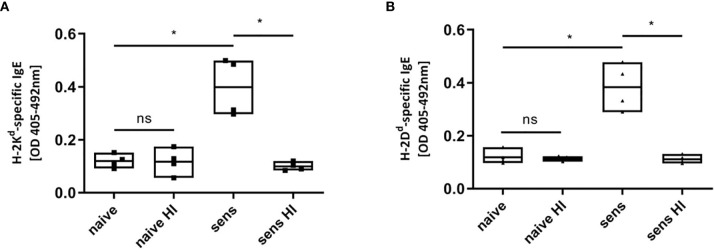
Heat inactivation of sensitized serum. MHC-monomers (H-2K^d^, **A**; H-2D^d^, **B**) were incubated with heat inactivated serum, destroying potential existing IgE. Heat inactivation (HI) leads to a loss of signal of MHC-specific IgE in C57BL/6 mice sensitized (sens) with a BALB/c (haplotype d) skin allograft. Results from two independent experiments are shown. (n=4 for sensitized and naïve) P values were calculated with Mann-Whitney U test. *P < 0.05, ns = not significant, P> =0.05.

In contrast to IgG, free IgE in plasma can be inactivated *via* heat (43). To provide additional evidence that the positive signals detected in ELISA occur from MHC-specific IgE and are not due to background signals or anti-MHC IgG detected by cross-reactivity, serum from C57BL/6 sensitized with a BALB/c skin allograft was heat inactivated at 56°C for 30 min. Antibodies of the IgG isotypes were not destroyed *via* this procedure (43). As shown in **Figure 6**, positive signals for MHC-specific IgE vanish when monomers were incubated with heat inactivated serum. Signals for MHC-specific IgG_1_ were not lost in treated serum (data not shown). This further confirms the detection of IgE specific for donor MHC, ruling out positive signals due to unspecific binding or MHC-specific IgG.

### Demonstration of the Functional Activity of MHC-Specific IgE in Mice

**Figure 7:**
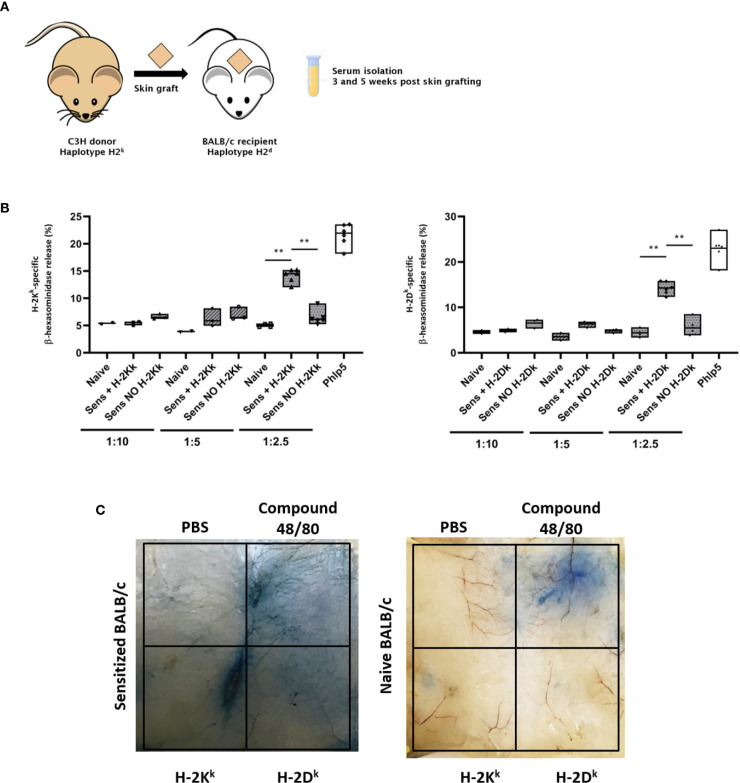
Testing of the functional activity of MHC specific IgE *in vitro* and *in vivo*. **(A)** Schematic illustration of the experimental set up. Skin of C3H donor mice was grafted onto BALB/c recipient mice. Serum was isolated 3 and 5 weeks after transplantation. **(B)** RBL assay for testing the functional activity of MHC-specific IgE *in vitro*. RBL cells were incubated with naïve and sensitized BALB/c serum, after rejection of a C3H skin graft (haplotype k), with or without the MHC class I monomer H-2K^k^ or H-2D^k^. Serum was diluted either 1:10, 1:5 or 1:2.5 with medium. Serum dilutions 1:10 and 1:5 were tested in one experiment (n=3). The experiment was repeated once with serum diluted 1:2.5 (n=6). P values were calculated with Mann-Whitney U test. **P< 0.01. **(C)** Assessment of functional activity of MHC-specific IgE *in vivo* using the cutaneous type I hypersensitivity assay. Evans Blue (0.5%) was injected i.v. into sensitized (with C3H skin) or naive BALB/c mice followed by an intra-dermal injection of MHC monomers. Mice were sacrificed and their skin was inverted. A blue discoloration of the inverted skin indicates mast cell degranulation due to the presence of MHC-specific IgE. This Figure shows a representative photo [see also (13)].

To demonstrate the functional activity of MHC-specific IgE *in vitro*, we adapted the RBL-2H3 assay, primarily used in allergy research. The cells used for this assay are a basophilic leukemia cell line derived from rats (RBL-2H3), binding murine IgE (44). Incubating these cells with the respective serum and the specific antigen of interest, they degranulate if the serum contains sufficient quantities of antigen-specific IgE, demonstrating the functional activity of the antigen-specific IgE. As the immune system of the BALB/c mouse strain has a bias towards type 2 responses producing more MHC-specific IgE after rejection of a fully mismatched allograft, we isolated serum from BALB/c mice sensitized with a C3H skin allograft (haplotype k) for the establishment of this method **(** **Figure 7A**) (45).

RBL-2H3 cells were incubated with serum, followed by incubation with MHC monomers. Degranulation was measured according to release of β-hexasomindase as fluorescence intensity. Percentage of degranulation was calculated according to the fluorescent value of 100% release from control cells achieved by treatment with Triton X100 (**Equation 1**). As positive control of specific basophil degranulation, serum from mice sensitized with a major grass pollen allergen (Phl p 5) was used. No significant difference in degranulation compared to naïve serum was achieved with serum dilutions from 1:10 to 1:5 (**Figure 7B**). However, we could show a significant difference in degranulation in sensitized serum, compared to cells incubated with naïve serum by using the same serum dilution also used for the MHC-specific ELISA (1:2.5) (**Figure 7B**). This demonstrates that this assay can be used to measure the functional activity of MHC-specific IgE *in vitro*.

A limiting factor of these functional assays is the large amount of serum (40 µl per tested well) needed for the detection and functional analysis of MHC-specific IgE *in vitro*. Therefore, it might be necessary to pool serum either from different mice of one group or serum from different time points of the same mouse. As serum levels of MHC-specific IgE in the murine model used here seem stable from week 5 after rejection until 12 months later, the latter option was feasible, but in other models stability of IgE levels over time might differ.

Adaptions in terms of serum concentrations should be taken into consideration when using these protocols in different mouse strains. Due to the high concentration of donor specific antibodies of the IgG isotypes in the used serum, it might come to saturation of the presented epitopes *via* possible epitope sharing of IgG and IgE. We do not recommend changing the monomer concentration, but it is possible to first test different serum concentrations when using these assays on other mouse models.

To assess the functional activity of MHC-specific IgE *in vivo*, we adapted the cutaneous type I hypersensitivity assay used in allergy research to measure anaphylaxis in mice (35). BALB/c mice were sensitized with fully mismatched C3H skin allografts. After rejection, the presence of MHC-specific IgE was verified using the MHC-specific ELISA. The mice were then injected i.v. with 0.5% Evans Blue, followed by an intra-dermal injection of MHC monomers and were then sacrificed. A blue discoloration of the skin indicated an increased vascular permeability due to the release of vasoactive mediators *via* mast cell degranulation (**Figure 7C**). This indicates the presence of mast cell-bound MHC-specific IgE and subsequent crosslinking *via* the specific MHC monomers and demonstrates the functional activity of DSAs of the IgE isotype *in vivo*. Compound F48/80 was used as positive control, leading to mast cell degranulation without the need of specific antigens. PBS was used as negative control. No degranulation took place in a naïve BALB/c mouse (as shown in **Figure 7C**), proving the specificity of this hypersensitivity assay.

### Detection of HLA-Specific IgE in Human Serum

To screen for HLA specific antibodies in human serum, a bead-based multiplexed immunoassay system was used. Beads expressing defined single HLA class I and class II antigens were employed (LABScreen Single Antigen HLA Class I/II, One Lambda, West Hills, CA, USA). Each kit contains about 100 different single antigen-coated beads to maintain wide coverage of different HLA specificities present in the population. Routine laboratories are screening mainly for the HLA- specific IgG subtype and are using standardized 5 µl of beads according to manufacturer’s instructions. In order to optimize the protocol, we performed a downscaling experiment of the beads with the goal of maintaining the same sensitivity but using lower amounts of beads. We used human serum of two highly sensitized kidney transplant recipients and the serum of one healthy male control who never received an organ transplant or a blood transfusion.

In order to test the optimal bead volume, two experiments were performed for each HLA class I and class II. Maintaining a ratio of 1:4 beads to serum, we scaled the beads to 1.5, 2, and 5 µl. Downscaling of the beads for HLA class I and II is shown in **Supplementary Figure 1** performed in two individual experiments. Positive bead reactivities are indicating the total number of IgE reactivities.

For our downscaling experiments, we used the MFI of each single bead, subtracted the background from each bead value and compared it to the background-subtracted MFI of one healthy control serum. For a positive classification this bead value had to be higher than the respective value of the healthy control. Additionally, a minimum requirement for a positivity was a MFI of >25 (13).

The change of bead reactivities and specificities is shown in **Supplementary Figure 1**, for the two patients for each HLA class I and II. Patient 1 exhibits for example in summary 37 positive reactivities by using 5 µl of beads, 45 reactivities by using 2.5 µl and 42 positive signals by using 1.5 µl bead suspension for HLA class I. In comparison to HLA class II, with 25, 33, and 33 positive signals while using either 5, 2.5, or 1.5 µl of beads. Patient 2 has 42, 46 and 41 signals in the downscaling experiment for HLA class I and 20, 24 and 27 for HLA class II. Summarizing, the differences by counting positive bead reactivities between using 1.5, 2, or 5 µl of beads are displayed in the table of **Supplementary Figure 1**. Moreover, bead count could be maintained in a range of 40 to 100 for each bead independently of using either 1.5, 2, or 5 µl. Overall, by using the manufactures washing buffer the background could be reduced therefore negative control serum exhibited mainly MFIs < 10.

Each HLA kit contains a negative control bead (bead #1), which is not coated with any antigen and demonstrates the background of the assay, and also a positive control bead (bead #2) which is coated with human IgG.

We analyzed the MFIs of the two positive IgE sera signals and compared positive signals for each downscaling attempt.

A strong and robust bead signal was observed by using 1.5 µl of beads with a bead count of 40 to 100/single antigen bead which led us to the conclusion that downscaling to 1.5 µl beads is sufficient and still effective for HLA- specific IgE Luminex measurements.

**Figure 8:**
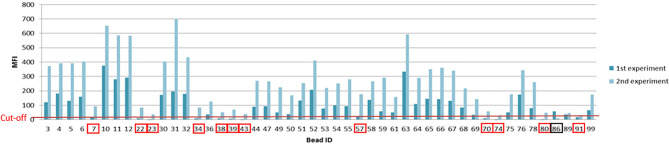
Differences of MFI profiles and antigen specificity within one patient from two individual Luminex based experiments. Changes in antigen specificities due to a difference in bead count are indicated by the red and black boxes (red= additional antigen specificity, black= loss of antigen specificity, from the first to the second experiment).

In the first experiment we had a bead count of >40 beads compared to the second experiment with less than 40 counts/bead resulting in 13 changed bead specificities (a plus of 12 positive signals and a loss of one signal). The changes of positive antigen specificities from those two experiments are shown in **Figure 8** and elucidate the importance of a robust bead count between experiments.

As human IgE is relatively unstable and sensitive to heat, samples should not be thawed more than two times before measuring on the Luminex instrument. Therefore, serum samples need to be stored in small aliquots at −80°C.

It is also necessary to vortex the bead suspension very well before starting to pipette them into the V-bottom plate. While using 1.5 µl of the bead suspension, it is essential to work very precisely and to resuspend the stock again after pipetting about 10 samples to maintain homogenous bead distribution as they tend to sediment. Furthermore, in order to preserve an acceptable bead count, after centrifugation the supernatant of each washing step needs to be removed with a pipette instead of inverting and flicking the plate.

**Figure 9:**
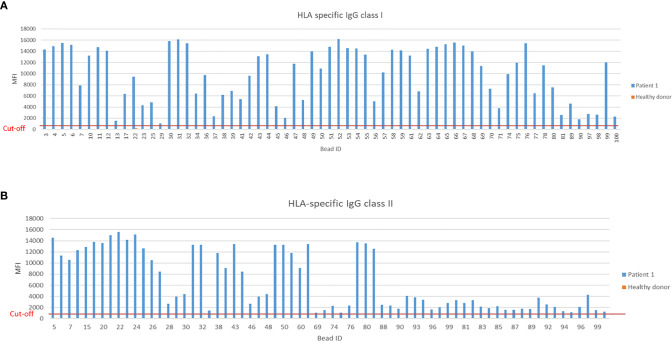
Measurement of HLA-specific IgG HLA class I and II in a sensitized kidney transplant recipient. **(A)** Positive MFI signals measured by Luminex bead based assay for IgG specific HLA class I and **(B)** HLA class II from a highly sensitized kidney transplant patient. Threshold for HLA-specific IgG reactivities was set at MFI>1000.

The single-antigen bead Luminex assay is routinely used in clinical diagnostic laboratories for testing sensitization of the recipient towards the donor prior to transplantation and to check for the development of *de novo* DSA post-transplantation. This assay is a very sensitive method based on the principle of flow cytometry which we modified for measuring IgE DSA. In **Figure 9**, the positive MFI of HLA-specific IgG reactivities class I of Patient 1 compared to the mean MFI of three healthy donors is shown. In summary, 65 positive HLA-specific IgG class I and 68 positive class II signals were detected with a cut-off set to MFI>1000.

Functionality of HLA-specific IgE antibodies was assessed in a basophil release assay and we could successfully demonstrate that IgE triggered basophils release and confirmed IgEs unique effector mechanisms (not shown) (13).

## Discussion

We established different methods for measuring donor specific IgE antibodies in the murine transplant setting and in human transplant patients. Although methods for measuring DSA in the murine setting are already available, they cannot directly discriminate MHC specificities (46). Here we demonstrate the successful establishment of an antigen-specific ELISA to measure MHC-specific antibodies for either class I or II.

The establishment of these assays for measurements of MHC-specific antibodies in the murine transplant models was done with monomers of the MHC class I isotype. However, ensuing experiments on antibody-mediated rejection done by our group were regularly performed using monomers of MHC class I and class II antigens.

Sensitivity for detecting IgE was assessed through several experiments. An important issue was the occurrence of masking IgG. Therefore, it was especially important to experiment with different antigen concentrations on the solid phase in order to increase the sensitivity of this assay. This prevents the issue of MHC-specific IgG competing with IgE for binding sites. Signals for MHC-specific IgE were lost, when serum was treated with heat and inactivating temperature-sensitive IgE. Moreover, cross-reactivity of the detecting antibody with other isotypes was ruled out by an immunoblot assay [see Figure E2 from (13)]. This demonstrates the specificity of this assay for detecting MHC-specific IgE ruling out false positive signals due to background or high serum concentrations of MHC-specific antibodies of other isotypes. Furthermore, we could effectively demonstrate the functional activity of MHC-specific IgE *in vitro* in an adapted rat basophil leukemia cell degranulation assay by using monomers to trigger mediator release.

One major limitation of measuring MHC-specific IgE in mice is the large amount of serum which has to be used for these assays. We have already demonstrated previously that MHC-specific IgE is present in murine serum until at least 12 months after transplant rejection (13). Therefore, we could solve this restriction of serum by pooling mouse sera from the same animal at different time points, if needed.

Using these assays on other mouse models or with different modes of sensitization, we recommend to test different serum concentrations in order to achieve as little background noise as possible in combination with a proper signal for measurement of MHC-specific IgE.

The cutaneous hypersensitivity type I reaction assay was used to show the functional activity of IgE specific antibodies *in vivo* in sensitized BALB/c mice. Due to the light skin color of this mouse strain, the degranulation of mast cells in the skin shown by the blue discoloration could be presented clearly. Therefore, we used this strain for demonstration purposes. However, this method is also expected to work in other donor-recipient combinations.

Luminex-based bead assays are routinely used in clinics for HLA-specific antibody screening and we could successfully establish a method for detection of HLA-specific IgE (47). In this paper we demonstrate that by downscaling the bead volume from 5 to 1.5 µl it remains a sensitive method with a good bead count (40–100 counts/single antigen bead) for detecting HLA-specific IgE antibody in human serum of highly sensitized kidney transplant recipients. While IgE is the least abundant isotype antibody in the blood, compared to IgG which is 10,000 times higher concentrated, and expected MFIs signals are around 100–500, a reduction of the bead volume was not accompanied with signal reduction or loss (48). Since IgE is not heat stable in human serum, compared to IgG, samples are temperature sensitive and need to be stored at −80°C. In addition, aliquots are not to be thawed more than two times for HLA-specific IgE measurement with beads.

The heat sensitivity of IgE was used to demonstrate the specificity of this bead based assay (13, 43). Incubation of serum at 56°C for heat inactivation, destroys HLA-specific IgE antibodies and positive signals are lost, similar to observation from the murine setting (**Figure 6**). Whereas, positive signals from heat insensitive HLA-specific IgG are not affected.

We could show the functional activity of HLA- specific IgE *in vitro* by an adapted humanized RBL assay using beads presenting a selected single antigen against which the patients were sensitized (13). Basophil release after antigen stimulation either for HLA class I or II was observed in patients who had positive HLA-specific IgE signals of an MFI ≈40, measured by Luminex, but there was no stimulation by antigens with negative IgE Luminex signals [see Figure 6D and E8 from (13)]. Moreover, there was no basophil degranulation upon heat inactivation of the serum [see Figure E9 from (13)].

Using these methods, we detected MHC-specific IgE in highly sensitized kidney transplant patients and in various mouse strains after rejection of fully mismatched skin or heart allografts. Furthermore, we also showed, that MHC-specific IgE is functionally active *in vitro* using the rat basophil leukemia cell degranulation assay in the murine transplant setting and the humanized rat basophil leukemia cell degranulation assay with serum from sensitized transplant patients (13).

Clinical studies of HLA-specific IgE in transplant patients (kidney, heart, liver and lung) are ongoing which will give us more insight into the production and potential role of HLA-specific IgE in transplant patients. Furthermore, the mechanistical background and role of MHC-specific IgE in cardiac transplant rejection is studied at the moment using a mouse model of antibody mediated rejection (CCR5 KO) established by the group of Fairchild et al. (49, 50).

In conclusion, we established various assays to measure MHC-specific IgE and to demonstrate its functional activity in murine and human transplant recipients which might be a valuable tool for further studies in antibody mediated rejection.

## Study Approval

The study participants were recruited at the Vienna General Hospital/Medical University of Vienna. All human samples were collected after written informed consent. The study was approved by the ethics review board of the Medical University of Vienna (EK No. 267/2011, EK No. 1535/2016).

All murine experiments were approved by the Ethics and Animal Welfare Committee of the Medical University of Vienna and were performed in strict accordance with national and international guidelines of laboratory animal care. All animals received humane care in compliance with FELASA and ARRIVE and were approved by the Austrian Federal Ministry of Science, Research and Economy BMWF GZ: 66.009/0295-1I/3b/2011 and GZ 66.009/0230- II/3b/2011.

## Data Availability Statement

The raw data supporting the conclusions of this article will be made available by the authors, without undue reservation.

## Ethics Statement

The studies involving human participants were reviewed and approved by Ethics review board of the Medical University of Vienna (EK No. 267/2011, EK No. 1535/2016). The patients/participants provided their written informed consent to participate in this study. The animal study was reviewed and approved by Ethics and Animal Welfare Committee of the Medical University of Vienna (BMWF GZ: 66.009/0295-1I/3b/2011, GZ 66.009/0230- II/3b/2011).

## Author Contributions

AW, JM, and TW designed the research. AW, JM, AF, UB, NP, AC, MM, and SH performed the research. MW, BL, and RV contributed new reagents/analytic tools. AW, JM, AF, and MW analyzed the data. AW, JM, and TW wrote the paper. All authors contributed to the article and approved the submitted version.

## Funding

This work was supported by the Austrian Science Fund (FWF; projects TRP151-B19 and W1212 to T.W.), and with institutional support from TEVA Pharmaceuticals Europe (for the investigator-initiated study “HLA-specific IgE antibodies in organ transplantation”). AW is a recipient of a DOC Fellowship of the Austrian Academy of Sciences at the Department of Surgery, Medical University of Vienna (DOC/25556). In addition, the study was funded by the Danube Allergy Research Cluster by the country of Lower Austria. Rudolf Valenta is a recipient of a Megagrant of the Government of the Russian Federation, grant No 14.W03.31.0024. The funder bodies were not involved in the study design, collection, analysis, interpretation of data, the writing of this article or the decision to submit it for publication.

## Conflict of Interest

The authors declare that the research was conducted in the absence of any commercial or financial relationships that could be construed as a potential conflict of interest.

## Supplementary Material

Supplementary Figure 1 Luminex based measurement of HLA class I and II specific IgE in human patient serum. Human serum from two patients (Patient 1 and Patient 2) and a healthy donor was incubated with either 1.5, 2, or 5 µl of beads specific for HLA class I and II. **(A)** Positive IgE reactivities upon bead assay are shown for HLA class I **(B)** and HLA class II. **(C)** Average of positive reactivities from Patient 1 and Patient 2 for HLA class I and II upon using 1.5, 2, or 5 µl of beads, from two independent experiments. **(D)** Overview of all positive IgE bead reactivities from Patient 1 for HLA class I from the first experiment compared to the healthy donor serum. Data are expressed as MFI.
