# Supplementary figures and images for: Methods to Detect MHC-Specific IgE in Mice and Men
Source: Front Immunol. 2020 Dec 8;11:586856. doi: 10.3389/fimmu.2020.586856 (PMC7753192; doi:10.3389/fimmu.2020.586856)

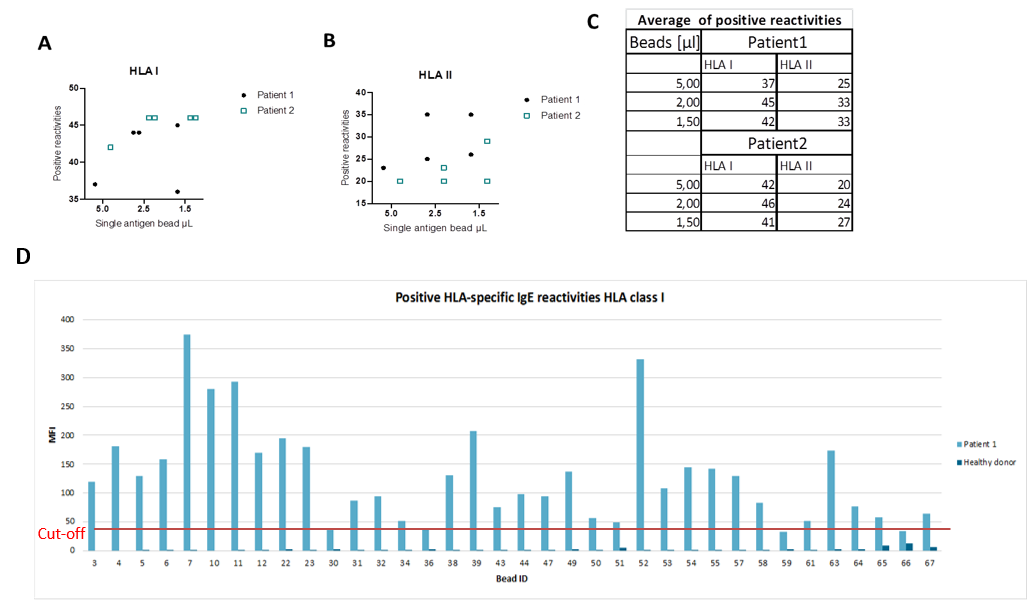

Supplement: Supplementary Figure 1 — Luminex based measurement of HLA class I and II specific IgE in human patient serum. Human serum from two patients (Patient 1 and Patient 2) and a healthy donor was incubated with either 1.5, 2, or 5 µl of beads specific for HLA class I and II. (A) Positive IgE reactivities upon bead assay are shown for HLA class I (B) and HLA class II. (C) Average of positive reactivities from Patient 1 and Patient 2 for HLA class I and II upon using 1.5, 2, or 5 µl of beads, from two independent experiments. (D) Overview of all positive IgE bead reactivities from Patient 1 for HLA class I from the first experiment compared to the healthy donor serum. Data are expressed as MFI. [file Image_1.tif]
